# Exploratory randomized pilot study of written diaphragmatic breathing instructions vs. a biofeedback device for upper abdominal pain

**DOI:** 10.1515/med-2026-1428

**Published:** 2026-05-11

**Authors:** Subhankar Chakraborty

**Affiliations:** Division of Gastroenterology, Hepatology and Nutrition, Department of Internal Medicine, The Ohio State University, Columbus, OH, USA

**Keywords:** abdominal pain, breathing exercise, diaphragmatic breathing, biofeedback, anxiety, depression

## Abstract

**Objectives:**

Conventional therapies for chronic upper abdominal pain are often ineffective. The objective of this exploratory pilot study was to investigate the effect of brief daily breathing exercises on pain severity.

**Methods:**

Adults were randomized to written diaphragmatic breathing instructions (WIDB) or a respiratory biofeedback device (Calmigo). Participants performed 3-min exercises thrice daily for 6 weeks. Pain severity (0–4 scale) was assessed weekly.

**Results:**

85 participants were randomized (WIDB n=36, Calmigo n=49). Linear mixed models showed significant pain reduction over time in both groups (β=−0.42 to −0.87, p<0.05), with no significant difference between groups (p=0.498). Exploratory analyses indicated that greater baseline pain (OR 2.49) predicted a higher likelihood of response, whereas White race (OR 0.40), diabetes (OR 0.48), and higher depression severity (OR 0.85) predicted a lower likelihood of response. No serious adverse events occurred.

**Conclusions:**

Brief daily breathing exercises, whether device-guided or self-guided, significantly improved upper abdominal pain. While no difference was detected between approaches, higher baseline severity predicted better response. Larger studies are needed to confirm these findings.

## Introduction

Upper abdominal pain is a common and often challenging symptom encountered in clinical practice. A study of nearly 25,000 people in the US found that approximately 81 % experienced abdominal pain in the past week, of whom the majority (61.5 %) sought medical care [1]. It is well known that chronic abdominal pain impairs one’s quality of life [2]. While pharmacologic therapies are frequently used to treat upper abdominal pain, they are often not effective in completely resolving it [3]. Non-pharmacologic interventions such as cognitive behavioral therapy and probiotics have shown some efficacy in abdominal pain-related functional GI disorders [4].

In recent years, diaphragmatic breathing has gained attention for its potential to modulate visceral pain through autonomic regulation and improved gut-brain signaling. It is a technique that emphasizes slow, deep breaths using the diaphragm and has been shown to reduce both acute and chronic pain [5], 6]. In the GI tract, it has been shown to reduce symptoms of rumination syndrome, irritable bowel syndrome, and excessive belching [7], [8], [9]. However, whether it helps those suffering from upper abdominal pain is unknown. Further, it is also unknown if the effectiveness of this intervention is affected by the method of instruction.

To date, few studies have directly compared the impact of instructional modality on the effectiveness of diaphragmatic breathing for symptom relief. A meta-analysis of randomized trials found that device-guided breathing (DGB) was as effective as a sham device on blood pressure in those suffering from hypertension [10]. A comparison of devices guided breathing to a sham device in patients suffering from post-traumatic stress disorder, however, found that DGB was superior to a sham device in reducing sympathetic reactivity to mental stress [11].

From the discussion above, we can clearly see a gap in our knowledge of whether breathing exercises affect the severity of upper abdominal pain. To address this gap, we conducted a prospective pilot study comparing the effect of breathing exercises on self-reported severity of upper abdominal pain. We hypothesized that daily brief breathing exercises could decrease the severity of upper abdominal pain. Our research question was whether two different strategies, namely written instructions on diaphragmatic breathing or a hand-held device, have a differential effect on self-reported severity of upper abdominal pain.

## Methods

This exploratory study was conducted at a tertiary university in the Midwestern United States. Participants were enrolled from across the United States. The protocol was approved by the Institutional Review Board at our University (Study ID: 2022H0256). To be eligible, participants had to be living in the United States, be at least 18 years old, understand English, have moderate-severe symptoms of anxiety or depression, and moderate-severe symptoms of dyspepsia (i.e., nausea, early satiety, postprandial fullness, or abdominal pain). Those who could not understand English were excluded. The rationale for our inclusion criteria has been described previously in previous manuscripts [12], 13]. These prior studies have explored the effect of breathing interventions on nausea and bloating, while the present study focuses on its effect on upper abdominal pain.

Participants rated the severity of their abdominal pain above the navel on a scale ranging from 0 (none) to 4 (Very severe) [14]. Anxiety and depression symptoms were assessed using the Hospital Anxiety and Depression Survey (HADS). Based on the HADS score, anxiety and depression symptoms were categorized as absent (0–7), mild [8], [9], [10], moderate [11], [12], [13], [14], or severe [15], [16], [17], [18], [19], [20], [21]. To be eligible, participants had to have at least moderate anxiety or depression (i.e., a HADS-anxiety or depression score of ≥11) [15].

After enrollment, participants completed a baseline survey about demographics. Then, they were randomized to either Calmigo^®^, a small handheld device, or to written instructions for diaphragmatic breathing. Calmigo^®^ guides users to extend their exhalation using multisensory cues (light, vibration, sound). The latter group received PDF instructions on how to perform diaphragmatic breathing exercises via email. The randomization was computer-generated and based on their age, sex, body mass index (BMI), and diabetes history. Blinding was not feasible due to the nature of the intervention. Investigators were aware of group allocation. The Calmigo^®^ group was encouraged to complete a 5-min training phone call with the Calmigo support team to supplement the written instructions for using the device. The written instruction group was not provided with any additional training beyond the instructions emailed to them. No additional support was provided to either group. Both groups were asked to practice breathing exercises thrice a day, 3 min at a time, for 6 weeks. At the end of each week, participants completed electronic surveys about their upper abdominal pain. The final survey was sent to participants one week after finishing the exercises (i.e. in week 7). We did not monitor adherence to the breathing exercises.

The overall goal of our study was to investigate the effect of breathing exercises on symptoms of gastroparesis. However, for this manuscript, we chose to focus our attention on the change in upper abdominal pain because it is a common and difficult-to-manage symptom. This was a *post hoc* analysis undertaken due to the research gap in this area.

Given the exploratory nature of our study and the limited availability of data on the effect of breathing exercises on upper abdominal pain, we did not perform a formal power calculation to determine the sample size for this study. Rather, the sample size was based on feasibility. Our goal was to have at least 40 participants in each group complete 6 weeks of the respective intervention. We estimated that the screen failure rate would be approximately 40 % because our criteria required both moderate to severe anxiety/depression and moderate to severe gastroparesis symptoms to be eligible. Further, we estimated that 60 % of those who were eligible would complete the consent form, and approximately 70 % of those who enroll will complete the baseline surveys that were required for randomization. Finally, we estimated that only a third of those who enter the intervention will complete the full six weeks of surveys. Based on this, we estimated that we will need to enroll up to 240 participants to reach our goal of having 40 participants in each group complete six weeks of the breathing interventions. All data was entered into REDCap. De-identified data were first downloaded as an Excel file and then analyzed using IBM SPSS version 29.0 (IBM Corporation, USA). A modified intention-to-treat analysis was conducted that included all those who completed the baseline survey about demographics.

Those participants who had at least 1 point improvement in abdominal pain severity at the end of week 4 compared to that at baseline were classified as “responders,” while those who had no change or worsening of abdominal pain were classified as “non-responders”.

Continuous data was expressed as a mean with standard deviation. Categorical data was expressed as frequency and percentage. Continuous data was compared between groups using an independent *t*-test (if there were 2 groups) or one-way ANOVA (if >2 groups).

**Primary Longitudinal Analysis:** A linear mixed model (LMM) was fitted to evaluate the effect of the intervention group and time on upper abdominal pain scores. The model included fixed effects for Group (Calmigo vs. Written Instructions), Time (treated as a categorical variable: Baseline [Week 0] through Week 7), and the Group × Time interaction. A random intercept was included for each participant to account for the repeated-measures nature of the data (correlation of scores within subjects). The “Calmigo” group and “Week 0” served as the reference categories.

**Supplementary within-person change (exploratory):** To aid interpretation of individual-level change, we conducted paired *t*-tests comparing each week to baseline within each group, reporting Cohen’s d and p-values adjusted for multiple comparisons using the Benjamini–Hochberg False Discovery Rate (FDR). These analyses are descriptive and exploratory and were not used for primary inference.

Categorical data was compared using the chi-square test. Univariate logistic regression was employed to examine how independent variables (mood, baseline symptom severity) and covariates (demographics, diabetes, BMI) predicted the dependent variable (response to breathing intervention with a ≥1 reduction in pain severity from baseline to week 4).

All results were expressed with 95 % confidence intervals where applicable. p-values were provided for all statistical tests. A p-value of less than 0.05 was considered statistically significant. Ethical approval was obtained from the Institutional Review Board at The Ohio State University (Study ID: 2022H0256). All participants provided informed consent before being enrolled in the study.

## Results

**Figure 1:**
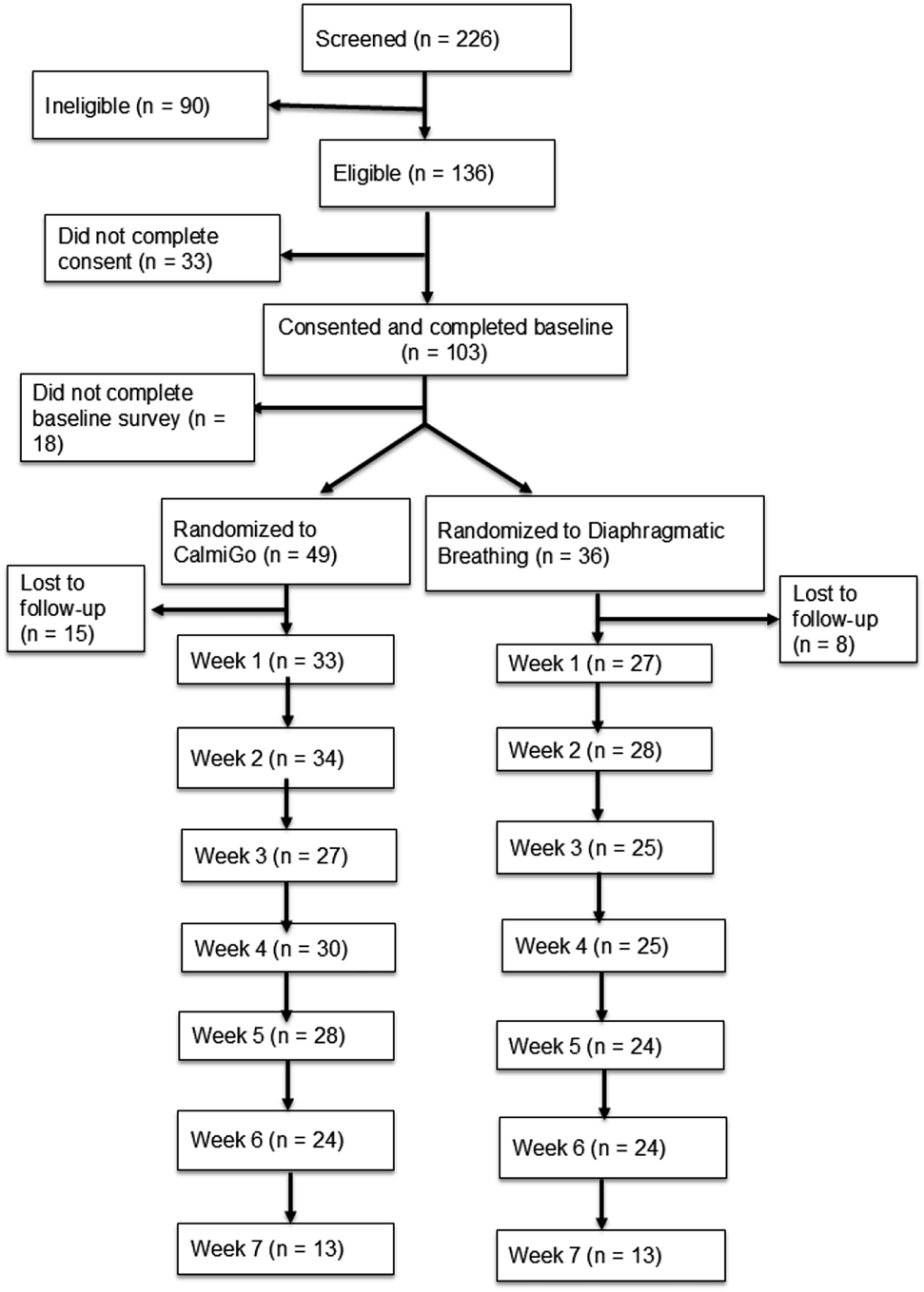
Consort flow diagram of participant progress through the study. A total of 226 participants were screened, of whom 90 were ineligible. Among 136 eligible participants, 33 did not provide consent and 18 did not complete the baseline survey, leaving 85 participants who were randomized (Calmigo: n=49; diaphragmatic breathing: n=36). Weekly retention numbers are displayed for each arm. Loss to follow-up occurred in 15 participants in the Calmigo arm and 8 in the diaphragmatic breathing arm. Reasons for loss to follow-up, where available, included voluntary withdrawal and inability to re-establish contact.

Figure 1 shows the participant enrollment and progression through the study. 226 people completed the screening surveys. Of the 136 who were eligible, 103 completed the consent form and 85 of them the baseline surveys. They were randomized to either device-guided breathing with Calmigo^®^ (n=49) or written instructions for diaphragmatic breathing exercises (n=36) based on their age, sex, BMI, and history of diabetes. 15 people in the Calmigo group and 8 in the Written Instructions group were lost to follow-up.

### Baseline characteristics

At baseline, 23 out of 49 (47 %) in the Calmigo group and 18 out of 36 (50 %) in the Written Instructions group reported severe or very severe abdominal pain. There was no difference in demographic characteristics between the two groups. There was also no difference in severity of anxiety or depression symptoms or in the proportion of diabetics. The two groups were also similar in the baseline severity of abdominal pain and in the proportion of patients with severe or very severe upper abdominal pain. Findings are summarized in Table 1.

**Table 1:** Comparison of baseline characteristics between Calmigo and Written Instructions group.

	Calmigo (n=49) Mean (SD)	Written Instructions Mean (SD), (n=36)	p-Value
Age (years)	41.08 (13.89)	43.47 (12.14)	0.411
White race	47	32	0.675
Females	39	32	0.169
BMI	28.37 (9.07)	27.35 (8.05)	0.601
HADS-anxiety	14.15 (2.92)	13.89 (3.56)	0.717
HADS-depression	9.89 (4.55)	11.03 (4.03)	0.240
Non-diabetics	40 (81.6 %)	34 (94.4 %)	0.158
Baseline upper abdominal pain severity	2.28 (1.2)	2.25 (1.32)	0.895
Proportion with severe or very severe abdominal pain	23 (47 %)	18 (50 %)	0.502

BMI, body mass index; HADS, hospital anxiety and depression scale; SD, standard deviation; p-value is for an independent sample *t*-test for continuous variables or the chi-square test for categorical variables.

### Change in abdominal pain with breathing exercises

**Figure 2:**
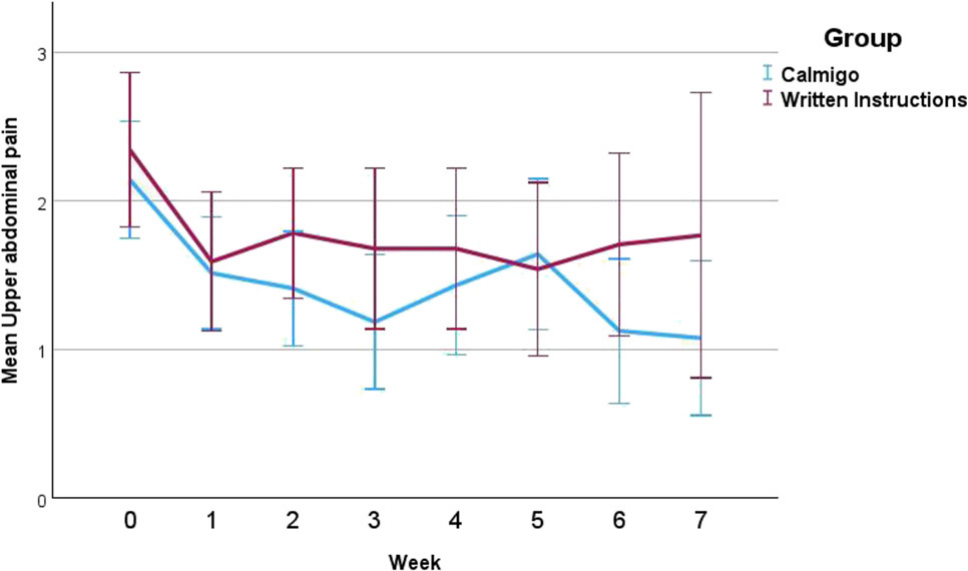
Mean (SE) upper abdominal pain severity over 7 weeks. Points represent means for the Calmigo and Written Instructions groups, with error bars indicating 95 % confidence intervals. Week 0 represents the baseline.

Figure 2 shows the average scores (with standard errors) for upper abdominal pain from baseline (week 0) to week 7 in the Calmigo and Written Instructions group. The linear mixed‑effects model analysis showed that, pain severity decreased significantly over time relative to baseline in the Calmigo group, which served as the reference group, with all follow‑up weeks (weeks 1–7) demonstrating significantly lower scores compared with baseline (all p < 0.05). However, there was no significant main effect of Group (β=0.21, SE=0.31, p=0.498), indicating that there was no overall difference in pain severity between the two groups. Furthermore, the Group × Time interaction was not statistically significant at any time point (all p>0.05) (Table 2) indicating that the magnitude of pain reduction over time was comparable between the Calmigo and Written Instructions groups.

Supplementary paired tests showed significant within-person decreases from baseline at several weeks in both groups (Supplementary Table 1), although these results should be interpreted cautiously due to multiple comparisons and their exploratory nature.

**Table 2:** Linear mixed model estimates for changes in upper abdominal pain severity over 7 weeks.

Parameter	Coefficient (β)	SE	z-statistic	p-Value	95 % CI lower	95 % CI upper
Intercept (Baseline Calmigo)	2.14	0.21	10.36	<0.001	1.74	2.55
Group [Written Instructions]	0.21	0.31	0.68	0.498	−0.39	0.81
Time effect (vs. Week 0)						
Week 1	−0.60	0.19	−3.08	0.002	−0.98	−0.22
Week 2	−0.75	0.19	−3.89	<0.001	−1.13	−0.37
Week 3	−0.87	0.21	−4.22	<0.001	−1.28	−0.47
Week 4	−0.68	0.20	−3.38	0.001	−1.07	−0.28
Week 5	−0.42	0.20	−2.05	0.041	−0.82	−0.02
Week 6	−0.85	0.22	−3.95	<0.001	−1.27	−0.43
Week 7	−0.79	0.27	−2.95	0.003	−1.31	−0.26
Interaction (Group × Time)						
Written Inst. × Week 1	−0.22	0.29	−0.75	0.454	−0.79	0.35
Written Inst. × Week 2	0.19	0.29	0.64	0.519	−0.38	0.75
Written Inst. × Week 3	0.20	0.30	0.65	0.518	−0.40	0.79
Written Inst. × Week 4	0.01	0.30	0.04	0.968	−0.57	0.60
Written Inst. × Week 5	−0.41	0.30	−1.36	0.174	−1.01	0.18
Written Inst. × Week 6	0.24	0.31	0.78	0.436	−0.37	0.85
Written Inst. × final survey	0.41	0.38	1.06	0.290	−0.35	1.16

Unstandardized regression coefficient (β), SE, standard error; z, z-statistic; p, two-tailed p-value; CI, 95 % confidence interval; The reference group is Calmigo and the reference time point is Baseline (Week 0); Analyses were adjusted for repeated measures using a random intercept for each participant.

### Influence of baseline characteristics on improvement in abdominal pain

#### Calmigo

Week 4 responders (≥1 point reduction in pain from baseline) had more severe abdominal pain (p=0.008), and depressive symptoms (p=0.002) at baseline than non-responders. They, however, did not differ from non-responders in demographic characteristics, BMI, HADS-anxiety score, or proportion of diabetics (Table 3).

**Table 3:** Comparing responders vs. non-responders after 4 weeks.

	Responders	Non-responders	p-Value
Calmigo			
n	15	15	
Age	43.8 ± 14.2	41.7 ± 15.4	0.69
White race	14 (93.3 %)	15 (100 %)	1.00
Females	12 (80 %)	13 (86.7 %)	1.00
BMI	25.4 ± 6.6	25.9 ± 7.4	0.84
Baseline abdominal pain	2.5 ± 0.99	1.5 ± 1.06	0.008
HADS-anxiety	13.1 ± 1.8	14.8 ± 3.4	0.092
HADS-depression	7.67 ± 3.35	12.46 ± 4.39	0.002
Diabetic	4 (26.7 %)	1 (6.7 %)	0.33
Written Instructions			
n	12	13	
Age	41.3 ± 8.93	43.8 ± 13.76	0.608
White race	10 (66.7 %)	13 (100 %)	0.22
Females	12 (80 %)	10 (66.7 %)	0.21
BMI	26.3 ± 9.36	26.1 ± 9.36	0.967
Baseline abdominal pain	3.08 ± 0.9	1.62 ± 1.56	0.009
HADS-anxiety	15.2 ± 3.35	12.7 ± 3.61	0.09
HADS-depression	10.92 ± 4.12	10.54 ± 3.26	0.80
Diabetic	0 (0 %)	2 (13.3 %)	1.00

#### Written instructions

Week 4 responders had more severe abdominal pain (p=0.009) at baseline than non-responders. However, there were no differences in demographics, BMI, HADS- anxiety or depression scores, or in the proportion of diabetics (Table 3).

### Factors associated with improvement in upper abdominal pain

For univariate logistic regression, we combined the week 4 responders and non-responders for both groups. The severity of upper abdominal pain at baseline was associated with a greater likelihood of improvement after 4 weeks of breathing exercises, while White race, being diabetic, and having more depressive symptoms were associated with a lower likelihood of response. Improvement in abdominal pain was independent of the type of breathing intervention (Table 4).

**Table 4:** Univariate logistic regression of factors associated with improvement in upper abdominal pain after 4 weeks.

	Odds ratio	95 % C.I.	p-Value
Age	1.00	0.99–1.01	0.95
White race^a^	0.40	0.23–0.69	0.001
Male sex	0.84	0.20–3.46	0.81
BMI	0.99	0.98–1.02	0.68
Diabetic^b^	0.48	0.28–0.84	0.009
Baseline severity of abdominal pain	2.49	1.44–4.32	0.001
HADS-anxiety	1.02	0.98–1.06	0.34
HADS-depression	0.85	0.81–0.89	<0.0001
Written instructions^c^	0.94	0.43–2.06	0.87

^a^Non-white race, ^b^non-diabetics, and ^c^Calmigo group were used as comparison.

## Discussion

In the present study, we found that breathing exercises improved the severity of upper abdominal pain. We did not detect a statistically significant difference in pain reduction between the written instruction and Calmigo groups. Being Non-White, non-diabetic, and having lower depressive symptoms at baseline were associated with a greater likelihood of improvement in upper abdominal pain after 4 weeks. Given the wide confidence intervals, further powered studies are required to determine if true differences exist between the two breathing interventions.

Managing chronic upper abdominal pain is challenging due to its multi-factorial etiology, frequent overlap with disorders of gut-brain interaction (DGBIs), and the limited efficacy and potential adverse effects of pharmacologic options. The American Gastroenterology Association recommends nonpharmacologic therapies like cognitive behavioral therapy and relaxation techniques, such as gut-directed cognitive behavioral therapy in the management of chronic functional abdominal pain [16], 17]. However, there is a nationwide shortage of psychologists who can provide gut-directed CBT, which makes this challenging to implement. Breathing exercises can be taught relatively easily and require little practice to learn, making them an attractive non-pharmacologic option to treat chronic health-related problems. However, data supporting their utility in upper abdominal pain is limited. Our results suggest that breathing exercises can alleviate upper abdominal pain, however the exploratory nature of the study and the relatively small sample size mean that the findings need to be validated in larger prospective studies.

The mechanisms by which breathing exercises improve pain remain to be fully elucidated. Experimental studies have found that deep breathing, especially at a slow and controlled pace, reduces stress and, through this mechanism, likely decreases visceral pain [18]. Breathing also improves ventilation efficacy, decreases air trapping and normalizes diaphragmatic function, decreases oxidative stress and inflammation, and has beneficial *psychological effects* such as enhancing relaxation, decreasing anxiety, and improving coping, which likely work together to mediate its beneficial effects [18], [19], [20].

Diaphragmatic breathing is also a potent regulator of the autonomic nervous system (ANS). It shifts the balance of the ANS from sympathetic arousal to parasympathetic dominance. This shift activates the vagus nerve, which in turn plays a crucial role in pain modulation. Vagal afferents inhibit nociceptive transmission at the spinal dorsal horn and reduce peripheral inflammation via the ‘cholinergic anti-inflammatory pathway.’ [21].

By dampening the inflammatory cytokines that sensitize visceral nociceptors, improved vagal tone has been proposed to act as a physiological ‘brake’ on visceral hypersensitivity, often a driver of functional abdominal pain [22].

Chronic abdominal pain is also often maintained by ‘central sensitization,’ which refers to the condition where the brain’s pain matrix (anterior cingulate cortex and insula) becomes hyper-responsive. Controlled slow breathing has been shown to synchronize brain activity with the respiratory cycle, specifically modulating the activity in the amygdala and insular cortex [23]. This effect in turn has been suggested to reduce the emotional impact of pain effectively ‘turning down the volume’ on visceral signals before they are perceived as pain [24].

Anatomically, the diaphragm shares fascial connections with the esophagus and stomach. Shallow, thoracic breathing common in chronic pain states, including upper abdominal pain, can potentially lead to a rigid diaphragm, which in turn increases intra-abdominal pressure. Deep diaphragmatic excursions, on the other hand, provide a rhythmic, gentle compression and mobilization of the abdominal viscera, reduce intra-abdominal pressure, improve visceral blood flow, and thus reduce visceral pain [25]. Future studies focused on patients with moderate to severe upper abdominal pain as the predominant symptom, combined with physiologic and biochemical testing, may help understand the mechanisms better.

Although limited in sample size, we examined predictors of response to breathing exercises in a subset of our participants. Those who achieved a ≥1-point decrease in pain from baseline on the 0–4 abdominal pain scale (25 % reduction) were considered responders. This proportional threshold is conceptually aligned with consensus recommendations that emphasize percentage change as the clinically interpretable unit for pain outcomes; in particular, IMMPACT notes that a ≥30 % reduction represents a moderately important improvement (with ≥50 % improvement being substantial) [26]. Although our 25 % cut-off is slightly below 30 %, it remains close to this benchmark and is further supported by anchor-based evidence in gastrointestinal pain, where an abdominal-pain reduction of ≥2.2 points on a 0–10 numeric rating scale (∼30 %) has been reported as being the minimum clinically important difference (MCID) in IBS cohorts [27]. In the absence of a validated MCID specific to our bounded 0–4 scale and target population, using a near-30 % proportional threshold is consistent with FDA PRO guidance that specifies responder definitions. While these considerations support the clinical interpretability of our responder definition and avoid overstating equivalence, we acknowledge that these findings need validation in more robustly powered prospective studies [26], 27].

Non-White race, non-diabetic status, and lower depressive symptom burden were associated with a greater likelihood of improvement in upper abdominal pain at the end of 4 weeks in our cohort. The association between pain and diabetes is supported by research in diabetic gastroparesis, which is often driven by autonomic neuropathy and frequently accompanied by more severe symptoms and a blunted symptom improvement relative to non-diabetics [28]. Likewise, the association between less severe depressive symptoms and better chances of pain reduction aligns with the well-described pain–depression dyad, in which depressive symptoms amplify pain perception and predict worse clinical trajectories in those suffering with gut–brain interaction disorders [29]. The association with race, however, should be interpreted with caution. Most of the existing suggests that non-White patients tend to have *worse*, not better, outcomes for pain [30]. Our observed pattern may therefore reflect context-specific factors (e.g., baseline severity, expectations, access during the trial, or regression to the mean) rather than a generalizable biological difference. These findings need to be examined in larger, prospectively powered cohorts.

A key limitation of our study was the lack of objective adherence monitoring. Without tracking the frequency and duration of home practice, we cannot accurately assess the dose-response relationship or determine if non-responders failed to improve due to lack of efficacy or lack of practice. Future confirmatory trials should incorporate robust adherence measures, such as digital daily logs (e-diaries) or passive monitoring via wearable respiratory sensors, to distinguish between intervention failure and non-adherence.

## Limits to study

Despite the encouraging effect of the intervention on abdominal pain, our study has several limitations. Our sample size was relatively small, and attrition rates were high, which limits the generalizability of the findings and reduces statistical power. We relied on self-reported symptom questionnaires, which can be associated with reporting bias. In the absence of long-term follow-up, we cannot assess whether the observed benefits were sustained over time. Finally, there is potential bias introduced by the fact that participants in the device group received device training and support, which was not available to those in the written-instruction group. Despite these limitations, our study provides evidence to suggest a role for brief breathing exercises to help with a challenging clinical problem.

Our study sample was intentionally restricted to individuals with moderate-to-severe anxiety and/or depression and gastroparesis-related symptoms, which limits the external validity of our findings. Although upper abdominal pain was analyzed as the primary outcome, participants did not represent a population with isolated upper abdominal pain. Instead, the cohort reflected a subgroup of patients in whom abdominal pain co-occurs with significant psychological comorbidity and symptoms related to gastroparesis. As a result, the observed responses to the interventions may not generalize to patients whose primary or only symptom is upper abdominal pain. The conclusions of this study should therefore be interpreted within the context of this specific clinical profile, rather than extrapolated to broader populations with isolated abdominal pain.

Finally, this study was not prospectively registered in a public clinical trial database. Consequently, the outcomes reported here, particularly the analysis of specific symptom sub-scales such as upper abdominal pain, should be interpreted as exploratory and hypothesis-generating rather than confirmatory. The lack of a pre-specified statistical analysis plan limits the ability to rule out selective reporting. Future studies aimed at replicating these findings must include prospective registration with clearly defined primary and secondary endpoints.

In summary, our study provides preliminary evidence that both written diaphragmatic breathing instructions and device-guided breathing may reduce upper abdominal pain. Further, response to these exercises appears to be affected by patient and baseline severity of pain. Larger, adequately powered trials with longer follow-up and objective tests are needed to confirm these preliminary findings.

## Supplementary Material

Supplementary Material
