# Supplementary material for: Exploratory randomized pilot study of written diaphragmatic breathing instructions vs. a biofeedback device for upper abdominal pain
Source: Open Med (Wars). 2026 May 11;21(1):20261428. doi: 10.1515/med-2026-1428 (PMC13157260; doi:10.1515/med-2026-1428)
Supplement: Supplementary file 1 — Supplementary Material [file j_med-2026-1428_suppl_001.docx]

Supplementary Table 1 Within-person change from baseline by week (paired t-tests)

| Time | N | Change from baseline | Effect size (Cohen’s *d*) | Paired t-test p-value | p-value after FDR correction | p-value Calmigo vs. Written instructions |
| --- | --- | --- | --- | --- | --- | --- |
| Calmigo |  |  |  |  |  |  |
| Δ Week 1 | 33 | -0.606 | -0.404 | 0.027 | 0.119 | 0.832 |
| Δ Week 2 | 34 | -0.765 | -0.597 | 0.001 | **0.039** | 0.620 |
| Δ Week 3 | 27 | -0.889 | -0.564 | 0.007 | 0.065 | 0.226 |
| Δ Week 4 | 30 | -0.567 | -0.418 | 0.030 | 0.118 | 0.570 |
| Δ Week 5 | 28 | -0.393 | -0.281 | 0.148 | 0.453 | 0.922 |
| Δ Week 6 | 24 | -0.708 | -0.531 | 0.016 | 0.101 | 0.728 |
| Δ Week 7 | 13 | -0.846 | -0.942 | 0.005 | 0.065 | 0.509 |
| Written instructions |  |  |  |  |  |  |
| Δ Week 1 | 27 | -0.852 | -0.659 | 0.002 | 0.057 |  |
| Δ Week 2 | 28 | -0.536 | -0.500 | 0.013 | 0.129 |  |
| Δ Week 3 | 25 | -0.600 | -0.520 | 0.016 | 0.129 |  |
| Δ Week 4 | 25 | -0.640 | -0.485 | 0.023 | 0.129 |  |
| Δ Week 5 | 24 | -0.750 | -0.479 | 0.028 | 0.131 |  |
| Δ Week 6 | 24 | -0.583 | -0.512 | 0.020 | 0.129 |  |
| Δ Week 7 | 13 | -0.538 | -0.387 | 0.188 | 0.585 |  |

N (Number of participants), FDR (false discovery rate).
